# DNA-Based Authentication and Metabolomics Analysis of Medicinal Plants Samples by DNA Barcoding and Ultra-High-Performance Liquid Chromatography/Triple Quadrupole Mass Spectrometry (UHPLC-MS)

**DOI:** 10.3390/plants9111601

**Published:** 2020-11-18

**Authors:** Marta Sánchez, Elena González-Burgos, Pradeep Kumar Divakar, M. Pilar Gómez-Serranillos

**Affiliations:** Department of Pharmacology, Pharmacognosy and Botany, Faculty of Pharmacy, Universidad Complutense de Madrid (UCM), 28040 Madrid, Spain; martas15@ucm.es (M.S.); elenagon@ucm.es (E.G.-B.); pdivakar@farm.ucm.es (P.K.D.)

**Keywords:** medicinal plants, DNA barcoding, *matk*, HPLC-MS method

## Abstract

There is growing interest for medicinal plants in the world drug market. Particularly, *Matricaria recutita* L., *Valeriana officinalis* L., *Tilia* spp., and *Camellia sinensis* (L.) Kuntze are some of the most consumed medicinal plants for treatment of minor health problems. Medicinal plants are seen as natural and safe; however, they can cause interactions and produce adverse reactions. Moreover, there is lack of consensus in medicinal plants regulation worldwide. DNA barcoding and UHPLC-MS technique are increasingly used to correctly identify medicinal plants and guarantee their quality and therapeutic safety. We analyzed 33 samples of valerian, linden, tea, and chamomile acquired in pharmacies, supermarkets, and herbal shops by DNA barcoding and UHPLC-MS. DNA barcoding, using *matk* as a barcode marker, revealed that CH1 sold as *Camellia sinensis* was *Blepharocalyx tweediei*, and sample TS2 sold as linden belong to Malvales. On the other hand, UHPLC-MS analysis revealed the presence of bioactive compounds (apigenin-7-glucoside, acetoxy valerenic acid, valerenic acid, epigallocatechin, and tiliroside). However, none of samples met minimum content of these active principles (except for valerenic acid in VF3) according to the European Medicines Agency (EMA) and Real Spanish Pharmacopeia. In conclusion, this study revealed the need to incorporate DNA barcoding and HPLC-MS techniques in quality controls of medicinal plants.

## 1. Introduction

Medicinal plants constitute the basis of traditional and modern primary healthcare. Over 80% of the population, mainly of developing countries, depend on traditional and herbal medicine. Moreover, at least 25% of drugs in the modern pharmacopeia are derived from plants [1]. Furthermore, it is estimated that pharmacological activity has been evaluated in only 15% of all 300,000 plant species identified [2]. The consumption of medicinal plants for disease prevention and health promotion has increased significantly in the last two decades [3]. The reasons that explain the rise in therapeutic use of medicinal plants are several. They include natural tendency in population, erroneous perception of its safety, lower economic cost compared to conventional medicines, and polypharmacy [4,5]. The high demand for medicinal plants is reflected in the economic data of world market. The global trade in herbs was over USD 83 billion in 2012, being especially high in India, China, and Germany [6,7]. These medicinal plants are available in pharmacies, supermarkets, and herbal shops [4,8].

These medicinal plants must meet standards of quality, safety, and efficacy. In this context, pharmacopeia monographs include tests and acceptance criteria ranging from botanical identification, pharmacognostic evaluation, and chemical characterization with chromatographic methods to evaluate the quality of herbal medicines [9]. However, one of the biggest difficulties related to quality is that commercial medicinal plants are crushed or powdered, being problematic to identify phenotype or part of the plant [10]. The DNA barcoding technique is postulated as an effective tool to overcome limitations in the quality controls of commercial medicinal plants. DNA-based techniques consist of using short DNA sequences from standardized gene regions (rbcL, matK, and ITS2) [11,12,13]. One of the great advantages of DNA barcoding technique is that the result is not influenced by harvesting period, growth condition, environmental factors, and sample age, among other factors [14,15,16]. Moreover, high-performance liquid chromatography (HPLC) and mass chromatography (MS) techniques are widely useful to identify and quantify bioactive compounds found in medicinal plants [14]. Therefore, the correct botanical identification by DNA barcoding and the precise bioactive compounds determination by HPLC-MS constitute an integrated approach to guarantee quality and safety of market medicinal plants [17,18,19].

*Matricaria recutita* L. (chamomile), *Valeriana officinalis* L. (valerian), *Tilia* spp. (linden), and *Camellia sinensis* (L.) Kuntze (tea) are found among the most consumed medicinal plants. They are commonly acquired in pharmacies, herbal shops, and supermarkets. Chamomile flowers as infusions have a beneficial effect on digestion; valerian root in capsules are used for reducing anxiety and a nervous state and improving sleep; linden leaves as infusions reduce anxiety symptoms; tea leaves as capsule or infusion bring relief from digestive problems [20].

The aim of the present work is to apply DNA barcoding and UHPLC-MS methods as a tool to evaluate the quality of market samples of *Matricaria recutita*, *Valeriana officinalis*, *Tilia* spp., and *Camellia sinensis.*

## 2. Results and Discussion

### 2.1. DNA Barcoding Analysis

Commercial herbal products can be adulterer, replaced, or suffer some kind of contamination [20,21]. Herbal products are sold as ground or powdered form of a raw herb, which makes correct botanical identification difficult [22]. DNA barcoding constitutes a very useful tool for quality control and, consequently, for clinical safety [23,24]. Molecular analyses is crucial for accurate and fast identification of medicinal plants, since the plant fragments sold in the market is difficult to identify using traditional methods especially due to lack of morphological features. Moreover, they often mixed with other plant materials, and in such cases, molecular analysis is one of the best approaches for accurate sample identification [25,26].

In this study, the DNA barcode marker *matk* was used to identify four of the most consumed medicinal plants, which were *M. recutita, V. officinalis, Tilia* spp., and *C. sinensis* [27,28,29]. A total of 33 market samples (pharmacies, herbal shops, and supermarkets) were investigated (nine samples from *M. recutita* flowers, seven from *V. officinalis* root, nine from *Tilia* spp. Leaves, and eight from *C. sinensis* leaves). DNA was successfully extracted from 23 of 33 markets samples. Raw material conditions (i.e., very dry, storage quality) could explain why DNA could not be extracted in 10 of the samples. A total of eighteen new *matk* sequences were generated for 23 samples isolated from chamomile, linden, tea, and valeria acquired in different distribution channels. These *matk* sequences were aligned with 38 sequences downloaded from GenBank. A total of 10 sequences were in *C. sinensis* data matrix, 20 in *M. recutita*, nine in *V. officinalis*, and 17 in *Tilia* spp. The length of all newly generated *matk* sequences was above 700 bp. All new sequences generated for this study have been deposited in GenBank.

**Figure 1:**
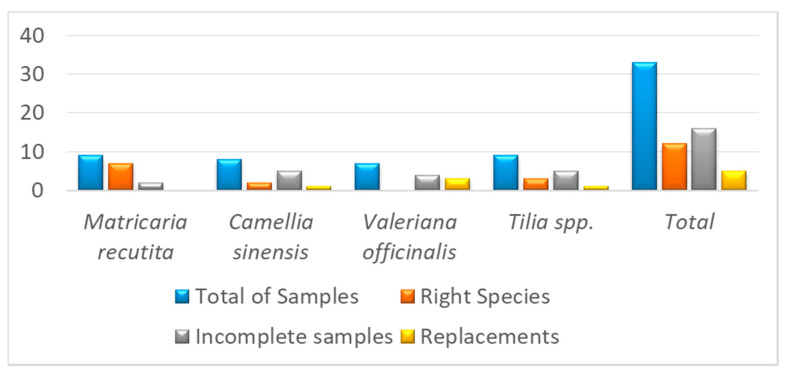
DNA-sequence-based identification of the analyzed medicinal plants marked samples.

The results of the molecular phylogenetic and DNA barcoding analyses were largely congruent. In the phylogenetic analysis, all samples were grouped together forming monophyletic clades except for CH1, TS2, and VH2 samples (Figures S1–S4). The authenticity of these samples was confirmed with DNA barcoding analysis using the Barcode of Life Data (BOLD) system. The results of the BOLD system blast are depicted in Figures S5–S8. The sample codes MH1 to MH3, MF2, and MS1 to MS3 were identified as *M. recutita*; CH2 and CF3 as *C. sinensis*; VH1, VS1, and VF2 as *Valeriana hirtella* Kunth.; TH1 to TH3 resulted *Tilia cordata* Mill. (Table 1). The sample CH1 sold as *C. sinensis* did not correspond to this species but to *Blepharocalyx tweediei* [30] Berg. *Blepharocalyx tweediei* is a tree native to Argentina and Uruguay that is traditionally used as infusion for cough, bronchospasm, diarrhea, and other intestinal disorders. Further, TS2 sample sold as Linden matched with Malvales; no closely related species was detected. Remarkably, samples sold as *V. officinalis* and *Tilia platyphyllos/europea* Scop. were identified as *Valeriana hirtella* Kunth. and *T. cordata*, respectively. On the other hand, all samples sold as chamomile were correctly identified as *Matricaria recutita*. Therefore, our study has identified alterations in *Camellia sinensis* and *Tilia* spp. in one of the three and one of the four samples, respectively, of these two genera belonged to entirely different species/order. Figure 1 shows the DNA final barcode identification of the analyzed marked medicinal plants. A percentage of 36.4 of plant samples were identified as right species, 48.5% were incomplete samples, and 15.2% were consider as replacements. This work reveals the need for a correct botanical identification for valerian and linden to ensure the precise labeling. DNA-based sample authentication is necessary for market medicinal plants for correct identification [13,31,32].

### 2.2. UHPLC/MS Analysis

**Figure 2:**
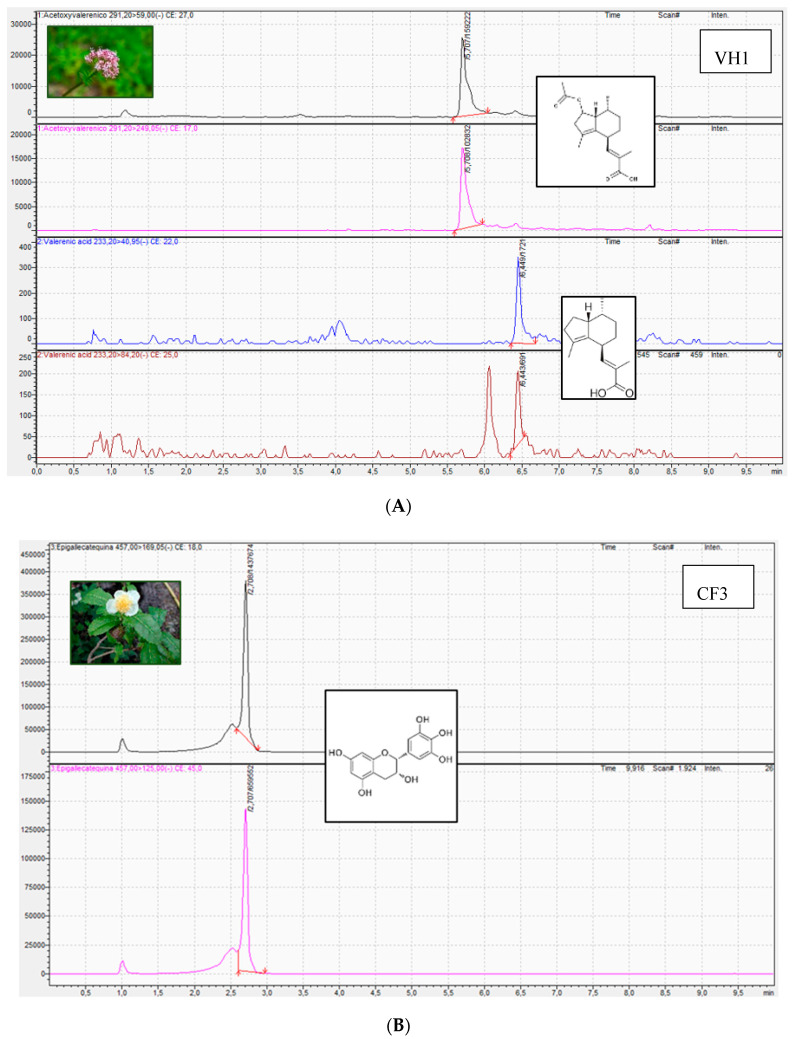

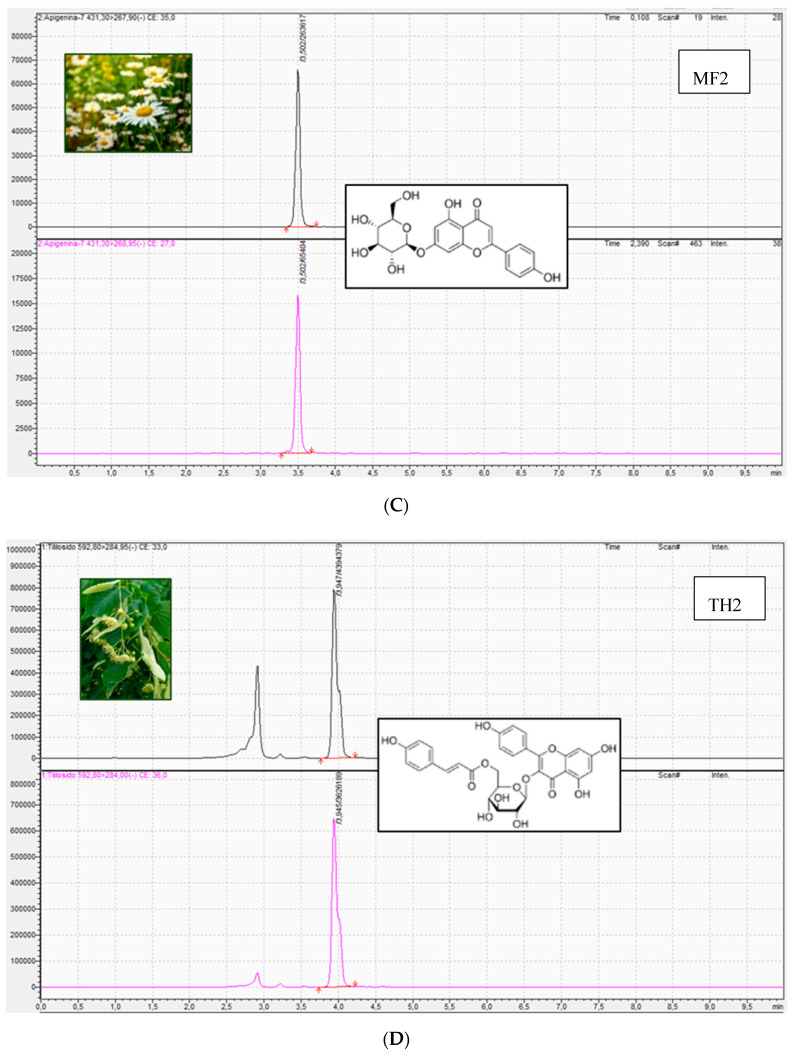
Representative UHPLC-ESI-QqQ-MS/MS chromatograms for (**A**) acetoxyvalerenic and valerenic acids presented in valerian samples, (**B**) epigallocatechin presented in tea samples, (**C**) apigenin presented in chamomile, and (**D**) tiliroside presented in linden.

UHPLC-MS based metabolomics approach allows to qualitatively and quantitatively analyze all metabolites in medicinal plant species with high sensitivity and precision [33]. The first of these techniques (molecular assays) can only authenticate the medicinal plant, while the second assesses (chromatographic assays) its quality and can provide information on the presence and concentration of compounds with pharmacological activity, but can never determine the identity of the species in question (providing no direct evidence of fraud). The simultaneous use of both techniques is therefore an additional advantage for the evaluation of the quality of medicinal plants and thus for their efficacy and safety. The 33 market samples of the medicinal plants *M. recutita*, *V. officinalis*, *Tilia* spp. and *C. sinensis* were analyzed by HPLC-MS to identify and quantify the main bioactive compounds responsible for their pharmacological activity [34] (Table 2, Table 3, Table 4 and Table 5, Figure 2). Particularly, the compound apigenin-7-glucoside was identified in *M. recutita* samples. The concentration of apigenin-7-glucoside ranged from 0.001 to 0.035%. Most of the samples acquired in the different commercial establishments had an average content of this active principle of 0.003%. Only two commercial samples of chamomile had a higher content in apigenin-7-glucoside, specifically MH1 sample acquired in an herbal shop (0.035%) and MS3 sample acquired in a supermarket (0.016%). The European Medicines Agency and the Real Spanish Pharmacopeia establish that apigenin-7-glucoside content should be at least 0.25% of dried drug [35,36], thus being the content in this bioactive compound lower in all analyzed samples. Moreover, previous studies have identified that the content for apigenin-7-glucoside in dry material of *Matricaria chamomilla* varied from 210 to 1110 mg/100 g using UPLC-UV method [37].

For *V. officinalis* samples, the bioactive compounds identified were acetoxy valerenic acid and valerenic acid. Acetoxy valerenic acid concentration ranged from 0.020 to 0.053%. The average content for this compound for all samples (except for VF3) was 0.025%. The content in acetoxy valerenic acid for samples VF3 acquired in pharmacy was 0.053%. On the other hand, a variable content of valerenic acid was identified among the different valerian samples (ranging from 0.048 to 0.167%). Hence, the content of this bioactive compound was similar in those samples from herbal shops (from 0.101 to 0.115%) and very different among pharmacy samples (0.048, 0.078, and 0.167%). On the other hand, the content of valerenic acid in the valerian sample from supermarket was 0.084%. According to the European Medicines Agency and the Real Spanish Pharmacopeia, the content in sesquiterpeneic acids should be not less than 0.17% expressed as valerenic acid [36,38]. The only sample that meets this requirement is VF3; this sample acquired in pharmacy could not be identified in the DNA barcoding study. Moreover, Navarrete et al. (2013) analyzed valerenic acid content in valerian species using liquid chromatography with ultraviolet detection. Particularly, the content of valerenic acid was 0.88% [39].

The compound epigallocatechin was identified and quantified in *C. sinensis* samples. Its content varied between 1.51 and 4.72%. The lowest content corresponded to a pharmacy sample and the highest content to an herbal shop sample. The most common average content identified in four of six samples from pharmacies and supermarkets was 2.39%. On the other hand, the epigallocatechin content of samples from supermarkets was very similar (3.29 and 3.71%). The content in flavan-3-ols including epigallocatechin should range from 10–25% of dried drug according to European Medicines Agency [40,41]. Previous works reported that the content in epigallocatechin was 4.62% in *Camellia sinensis* samples [42].

Finally, tiliroside was identified in tea samples with a content variation between 0.008 and 0.043%. The low content of 0.008% was quantified in an herbal shop sample. The rest of linden samples had an average of 0.034%. There are no data on tiliroside content neither in the European Medicines Agency nor in the Real Spanish Pharmacopeia [43]. The content of tiliroside has been identified to be higher in inflorescences that in leaves in *Tilia cordata* (49.2 µg/g versus 16.1 µg/g) [44].

The analysis of the secondary metabolites revealed that all the plant species analyzed, regardless of whether or not they had been correctly identified botanically, contained to a greater or lesser extent the bioactive compounds that were sought. This shows that these compounds are presented in other species of the same genus (i.e., *Valeriana hirtella*) and of other orders (i.e., Malvales). Specifically, the tiliroside has been identified in many different families including Malvaceae and Tiliaceae [44].

## 3. Materials and Methods

### 3.1. Reagents

Methanol HPLC grade was obtained from Panreac Química (Barcelona, Spain). Acetonitrile HPLC grade, formic acid HPLC grade, SYBR safe DNA gel stain, and the primers MatK-1RKIM-f and MatK-3FKIM-r were from Thermo Fisher Scientific (Runcorn, Cheshire, UK). Agarose MB 250 was purchased from Biotools Biotechnological and Medical Laboratories SA (Madrid, Spain). Apigenin-7-glucoside, epigallocatechin, and tiliroside were acquired from Extrasynthese (Genay Cedex, France). Acetoxy acid was from Sigma-Aldrich (St. Louis, MO, USA), and valerenic acid was from Chromadex (Irvine, CA, USA).

### 3.2. Herbal Products

The 33 samples of the four medicinal plants *Matricaria recutita* L., *Valeriana officinalis* L., *Tilia* spp., and *Camellia sinensis* (L.) Kuntze were acquired from supermarkets, herbal shops, and pharmacies located in the Autonomous Community of Madrid (Spain). Particularly, nine of these samples were from *M. recutita* flowers (3 samples from pharmacies, 3 samples from herbal shops, and 3 samples from supermarkets)*,* seven from *V. officinalis* root (3 samples from pharmacies, 3 samples from herbal shops, and 1 sample from supermarkets)*,* nine from *Tilia* spp. leaves (3 samples from pharmacies, 3 samples from herbal shops, and 3 samples from supermarkets), and eight from *C. sinensis* leaves (3 samples from pharmacies, 3 samples from herbal shops, and 2 samples from supermarkets) (Table 6). They were stored in conditions of temperature and ambient humidity.

### 3.3. DNA Barcoding Analysis

#### 3.3.1. DNA Extraction

DNA was extracted from market medicinal plants samples of roots (*V. officinalis*), leaves (*Tilia* spp. and *C. sinensis*), and flowers (*M. recutita*) using the Speed Tools tissue DNA extraction kit Biotools Biotechnological and Medical Laboratories following manufacturer’s instructions. Approximately, 100 mg of samples were pulverized with a sterile mortar in liquid nitrogen at room temperature and secondary metabolites were eliminated with methanol before starting DNA extraction. Then, samples were soaked in methanol for 2 h to remove potential secondary metabolites and dried overnight. Later, samples were incubated into lysis buffer initially at 65 °C for 30 min. In addition, root material samples were kept at room temperature overnight. After DNA extraction, samples were revealed in a 1% agarose gel stained with SYBR safe to check DNA quality [18].

#### 3.3.2. PCR and Sequencing

*Matk* was chosen in this study, since it is one of the universal DNA barcode markers for land plants [25]. The matK gene of chloroplast is 1500 bp long, located within the intron of the trnK. Since this gene is larger in length, a fragment of this is used for DNA barcoding analysis. Further, the gene contains high substitution rates within the species and is a potential candidate for DNA barcoding studies [11]. PCR amplifications of *matk* were performed using specific primers MatK-1RKIM-f and MatK-3FKIM-r (Ki-Joong Kim, pers. comm.). The reaction mixture (25 µL final volume) contained 5 μL of DNA (1:10), 4.5 μL sterile water, 12.5 μL REDTaq ReadyMix PCR Reaction M, and 1.5 μL of each primer (forward and reverse) at 10 μM. PCR amplifications were carried out in a Techne R TC-3000 thermal cycler with the following conditions: one initial heating step of 45 s at 98 °C, followed by 35 cycles of 10 s at 98 °C, 30 s at 54 °C, and 40 s at 72 °C. A final extension step of 10 min at 72 °C was added, after which samples were kept at 4 °C.

Once PCR amplification is completed, a 1% agarose gel stained with SYBR safe was used to visualize DNA. PCR products were purified by Speed-Tools PCR Clean Up (Biotools Biotechnological and Medical Laboratories SA, Madrid, Spain) kit following the manufacturer’s instructions, and sequencing was performed with labelling using BigDye Terminator v. 3.1 Kit (Applied Biosystems, Madrid, Spain) as follows: 35 cycles of 20 sec at 94 °C, 20 sec at 48 °C, and 4 min at 60 °C. Sequences were obtained in an ABI PRISM 3130 Genetic Analyzer (Life Technologies, Alcobendas, Madrid, Spain). This is a standard approach to analyze PCR products.

#### 3.3.3. Data Analysis

First, DNA sequences were assembled and manually adjusted in BioEdit sequence alignment editor software (v 7.2). Then, a second edition and assembly of the sequence fragments was made with the program SeqMan v.7 (Lasergene R, DNASTAR, Madison, WI, USA). Sequence identity was assessed using the mega-BLAST search function in GenBank [45,46]. Sequences with equal and above 95% similarity were downloaded from the GenBank and aligned with the newly generated sequences for this study. Separate dataset for each taxa viz. *M. recutita*, *V. officinalis*, *Tilia* spp., and *C. sinensis* were prepared for phylogenetic analysis. Each dataset was aligned using MAFFT v.7 [47] implementing the G-INS-I alignment algorithm, “1PAM/K = 2” scoring matrix with an offset value of 0.0, and the remaining parameters set to default values. The ambiguous regions in the *matk* alignment were assessed and removed using the least stringent option in Gblocks v.0.91b [48]. The alignments were analyzed using the maximum likelihood approach with RAxML v.8.2.6 program [49] as implemented on the CIPRES Web Portal, with the GTRGAMMA model. Nodal support was evaluated using 1000 bootstrap pseudoreplicates. The maximum likelihood (ML) trees were mid-point rooted. Phylogenetic trees were drawn using FigTree v.1.4.2 [50]. Moreover, sample identification was also assessed using a genetic distance-based blast option on the Barcode of Life Data System (BOLD Systems v3) [51]. The default setting was used for BLAST algorithm of the standard BOLD identification engine for *matK* sequences.

### 3.4. UHPLC-MS/MS Analysis

Samples were previously pulverized with a mortar and pestle sterile. Thirty milligrams of each medicinal plant samples were dissolved in 1 mL of ethanol/water 70/30% (*v/v*). Then, chamomile, linden, and tea samples were diluted 1:5 or 1:500, and valerian samples were diluted 1:4. HPLC standards were prepared at a concentration of 20 mg/L in methanol HPLC grade. Dilutions were prepared in a range of 0.05 to 1 mg/L in ethanol/water 70/30% (*v/v*) [52,53].

An ultra-high-performance liquid chromatography coupled with triple quadrupole mass spectrometry technique (UHPLC-QqQ-MS/MS) was developed using a LC-QQQ 8030 equipment (Shimazdu, Tokyo, Japan). The column was Phenomenex Gemini 5u C18 110A, 150 × 2 mm (Phenomenex, Alcobendas, Spain). The gradient mode was 7 min 5–95% Phase B; 8 min 95% Phase B; 8.5 min 5% Phase B using acetonitrile; in Phase A 0.1% formic acid in water. The flow rate was 0.5 mL/min and the infection volume was 10 µl for all medicinal plant samples, except for valerian, which was an injection volume of 20 μL.

Regarding LC-MS analysis, the mass spectrometer electrospray capillary voltage was maintained at 4.0 kV and the drying gas temperature at 250 °C with a flow rate of 15 mL/min. Nebulizer working flow was set at 1.5 mL/min. Nitrogen was used as both nebulizing and drying gas. Detection was carried out in Multiple reaction monitoring (MRM) mode, with a dwell time of 100 ms by monitoring three selective transitions for each parent compound. MRM transitions and their collision energy (CE) are shown in Table 7. The sample injection volume was 10 μL.

Data are expressed as means ± standard deviations of triplicate independent analyses.

## 4. Conclusions

DNA barcoding is revealed as an effective and necessary tool in the identification and authentication of plant species that are used in therapeutics. Since DNA barcoding does not provide qualitative or quantitative information on the metabolites of the plant raw material, its use together with chromatographic techniques such as HPLC-MS allows us to determine the precise identification of the species and the metabolic profile with great sensitivity and precision. The HPLC-MS technique combines the high resolution of ultra-high-performance liquid chromatography with high-resolution MS for molecular quantification. This technique has shown a high efficiency and resolution, a short time analysis, and increased sensitivity. The current work showed a high success rate in obtaining PCR amplification and sequencing of *matk* locus for the accurate sample identification of the market samples of medicinal plants. DNA barcoding demonstrated that the labeling of some medicinal plants acquired in different distribution channels is incorrect, demonstrating the need to apply DNA barcoding methods in the quality control of herbal products to ensure correct botanical identification and therefore ensure product quality. Moreover, the UHPLC-MS analysis displayed that all the analyzed samples presented the bioactive compounds (apigenin-7-glucoside, acetoxy valerenic acid, valerenic acid, epigallocatechin, and tiliroside) responsible for the pharmacological activity. This content was very variable for some plant species, as is the case of valerenic acid in valerian. In addition, none of the analyzed samples (except for valerenic acid in VF3) met the minimum content of these active principles according to the European Medicines Agency and the Real Spanish Pharmacopeia. This study shows the need to incorporate leading-edge molecular and analytical techniques for the quality control of plant species that are used in therapeutics to guarantee patient safety.

Future trends in the field of medicinal plants should be aimed at conducting more quality control studies in other widely consumed species and incorporating the combination of these molecular and analytical techniques in the plant industry for therapeutic purposes and even incorporating them into official documents (Pharmacopoeia, EMA).

## Figures and Tables

**Table 1:** Sample identification using the Barcode of Life Data (BOLD) systems.

Sample Code	Labelled	BOLD System Identification
MH1	Matricaria	*Matricaria recutita*
MH2	Chamomile	*Matricaria recutita*
MH3	*Matricaria chamomilla*	*Matricaria recutita*
MF2	Chamomile	*Matricaria recutita*
MS1	Chamomile	*Matricaria recutita*
MS2	*Matricaria recutita*	*Matricaria recutita*
MS3	Chamomile	*Matricaria recutita*
CH1	*Camellia sinensis*	*Blepharocalyx tweediei*
CH2	Thea	*Camellia sinensis*
CF3	Thea	*Camellia sinensis*
VH1	*Valeriana officinalis*	*Valeriana hirtella*
VS1	*Valeriana officinalis*	*Valeriana hirtella*
VF2	*Valeriana officinalis*	*Valeriana hirtella*
TH1	*Tilia platyphyllos*	*Tilia cordata*
TH2	Linden	*Tilia cordata*
TH3	Tilia Europea	*Tilia cordata*
TS2	Linden	*Malvales*

**Table 2:** Content of apigenin-7-glucoside in *Matricaria recutita* in samples labeled as acquired in pharmacies, herbal shops, and pharmacies. Data are expressed as means ± standard deviations of triplicate independent analyses.

*Matricaria recutita* L.	
Sample	Apigenin-7-glucoside (mg/g) (mean ± SD)
MH1	0.35 ± 0.015
MH2	0.02 ± 0.004
MH3	0.05 ± 0.005
MS1	0.07 ± 0.004
MS2	0.06 ± 0.003
MS3	0.16 ± 0.009
MF1	0.03 ± 0.002
MF2	0.01 ± 0.004
MF3	0.03 ± 0.005

**Table 3:** Content of valerenic acid and acetoxyvalerenic acid in *Valeriana* in samples labeled as acquired in pharmacies, herbal shops, and pharmacies. Data are expressed as means ± standard deviations of triplicate independent analyses.

*Valeriana Officinalis* L.		
Sample	Acetoxyvalerenic Acid (mg/g) (mean ± SD)	Valerenic Acid (mg/g) (mean ± SD)
VH1	0.26 ± 0.030	1.09 ± 0.017
VH2	0.24 ± 0.011	1.15 ± 0.017
VH3	0.24 ± 0.014	1.01 ± 0.020
VF1	0.20 ± 0.020	0.48 ± 0.015
VF2	0.29 ± 0.017	0.78 ± 0.026
VF3	0.53 ± 0.014	1.67 ± 0.030
VS1	0.25 ± 0.055	0.84 ± 0.015

**Table 4:** Content of epigallocatechin in *Camellia sinensis* in samples labeled as acquired in pharmacies, herbal shops, and pharmacies. Data are expressed as means ± standard deviations of triplicate independent analyses.

*Camellia Sinensis* (L.) Kuntze	
Sample	Epigallocatechin (mg/g) (mean ± SD)
CH1	21.2 ± 0.025
CH2	47.2 ± 0.015
CH3	23.2 ± 0.060
CS1	32.9 ± 0.025
CS2	37.1 ± 0.011
CF1	15.1 ± 0.020
CF2	25.9 ± 0.011
CF3	25.5 ± 0.030

**Table 5:** Content of tiliroside in *Tilia* in samples labeled as acquired in pharmacies, herbal shops, and pharmacies. Data are expressed as means ± standard deviations of triplicate independent analyses.

*Tilia* spp.	
Sample	Tiliroside (mg/g) (mean ± SD)
TH1	0.32 ± 0.056
TH2	0.39 ± 0.045
TH3	0.08 ± 0.020
TS1	0.27 ± 0.026
TS2	0.42 ± 0.032
TS3	0.43 ± 0.025
TF1	0.23 ± 0.020
TF2	0.36 ± 0.010
TF3	0.35 ± 0.011

**Table 6:** Samples of the medicinal plants chamomile, valerian, tea, and linden acquired from supermarkets, herbal shops, and pharmacies located in the Autonomous Community of Madrid.

Chamomile			Valerian		
Pharmacies	Herbal Shops	Supermarkets	Pharmacies	Herbal Shops	Supermarkets
MF1	MH1	MS1	VF1	VH1	VS1
MF2	MH2	MS2	VF2	VH2	
MF3	MH3	MS3	VF3	VH3	
**Linden**			**Tea**		
**Pharmacies**	**Herbal Shops**	**Supermarkets**	**Pharmacies**	**Herbal Shops**	**Supermarkets**
TF1	TH1	TS1	CF1	CH1	CS1
TF2	TH2	TS2	CF2	CH2	CS2
TF3	TH3	TS3	CF3	CH3	

**Table 7:** Multiple reaction monitoring (MRM) transitions and t collision energy conditions.

Active Compound	Transition	CE	Dwell (msec)
Acetoxyalerenic acid	291.3 > 59.00	27 V	100
	291.3 > 249.05	17 V	100
Valerenic acid	233.2 > 40.92	22 V	100
	233.2 > 84.20	25 V	100
Epigallocatechin	457 > 169.05	18 V	100
	457 > 125.00	45 V	100
Tiliroside	592.8 > 284.95	33 V	100
	592.8 > 254.95	55V	100
Apigenin-7-*O*-glucoside	431.3 > 267.90	35 V	100
	431.3 > 150.95	52 V	100

## Supplementary Materials

The following are available online at https://www.mdpi.com/2223-7747/9/11/1601/s1, Figure S1: Phylogenetic tress for *M. recutita.* Figure S2: Phylogenetic tress for *V. officinalis.* Figure S3: Phylogenetic tress for *Tilia* spp. Figure S4: Phylogenetic tress for *C. sinensis.* Figure S5: BOLD System for *M. recutita.* Figure S6: BOLD System for *V. officinalis.* Figure S7: BOLD System for *Tilia* spp. Figure S8: BOLD System for *C. sinensis.*
