# Supplementary material for: DNA-Based Authentication and Metabolomics Analysis of Medicinal Plants Samples by DNA Barcoding and Ultra-High-Performance Liquid Chromatography/Triple Quadrupole Mass Spectrometry (UHPLC-MS)
Source: Plants (Basel). 2020 Nov 18;9(11):1601. doi: 10.3390/plants9111601 (PMC7698941; doi:10.3390/plants9111601)
Supplement: Supplementary file 1 [file plants-09-01601-s001.zip › Figure S7.pdf]

IDENTIFICATION ENGINE: RESULTS

Results Summary

Query ID	Best ID	Search DB
unlabeled_sequence	*Tilia cordata*	MATK_RBCL

Query: unlabeled_sequence
Top Hit: Malvales - *Tilia cordata*

Score Summary

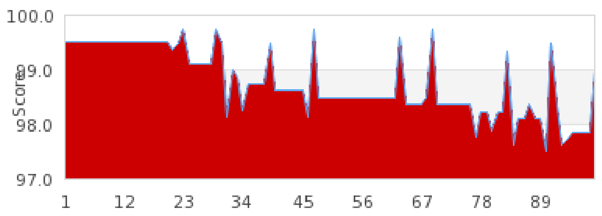

Scores indicate the degree of similarity between the query sequence and hits. Higher is better.

E-Value Summary

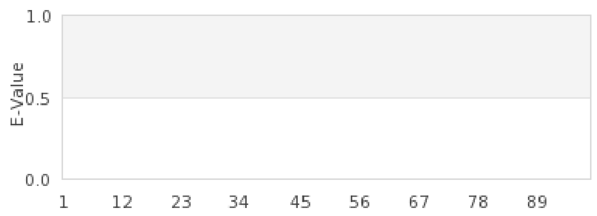

E-Values are an indicator of the likelihood that a given match was generated randomly. Lower is better.

IDENTIFICATION ENGINE: RESULTS

Results Summary

Query ID	Best ID	Search DB
unlabeled_sequence	*Tilia cordata*	MATK_RBCL

Query: unlabeled_sequence
Top Hit: Malvales - *Tilia cordata*

Score Summary

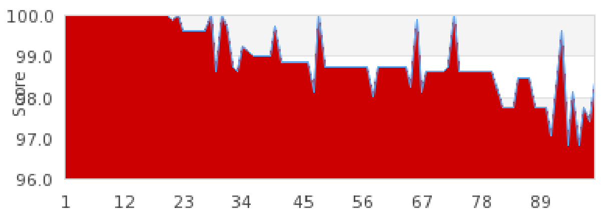

Scores indicate the degree of similarity between the query sequence and hits. Higher is better.

E-Value Summary

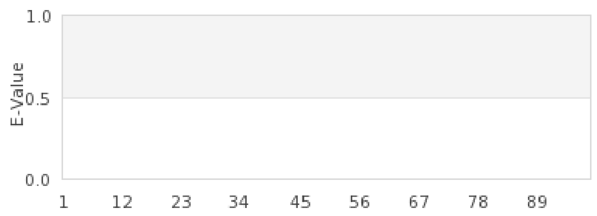

E-Values are an indicator of the likelihood that a given match was generated randomly. Lower is better.

IDENTIFICATION ENGINE: RESULTS

Results Summary

Query ID	Best ID	Search DB
unlabeled_sequence	*Tilia cordata*	MATK_RBCL

Query: unlabeled_sequence
Top Hit: Malvales - *Tilia cordata*

Score Summary

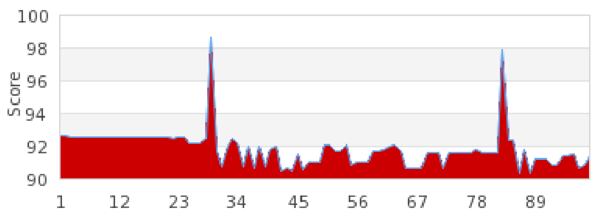

Scores indicate the degree of similarity between the query sequence and hits. Higher is better.

E-Value Summary

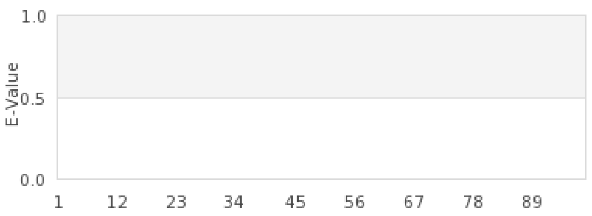

E-Values are an indicator of the likelihood that a given match was generated randomly. Lower is better.

IDENTIFICATION ENGINE: RESULTS

Results Summary

Query ID	Best ID	Search DB
unlabeled_sequence	*Malvales*	MATK_RBCL

Query: unlabeled_sequence
Top Hit: Malvales

Score Summary

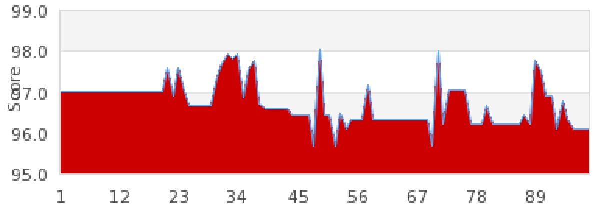

Scores indicate the degree of similarity between the query sequence and hits. Higher is better.

E-Value Summary

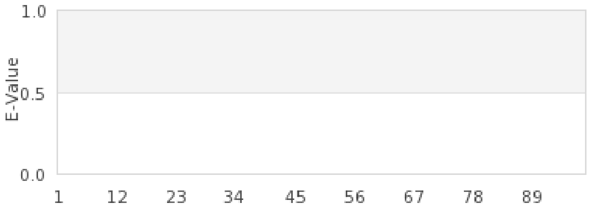

E-Values are an indicator of the likelihood that a given match was generated randomly. Lower is better.
