# Supplementary figures and images for: DNA-Based Authentication and Metabolomics Analysis of Medicinal Plants Samples by DNA Barcoding and Ultra-High-Performance Liquid Chromatography/Triple Quadrupole Mass Spectrometry (UHPLC-MS)
Source: Plants (Basel). 2020 Nov 18;9(11):1601. doi: 10.3390/plants9111601 (PMC7698941; doi:10.3390/plants9111601)

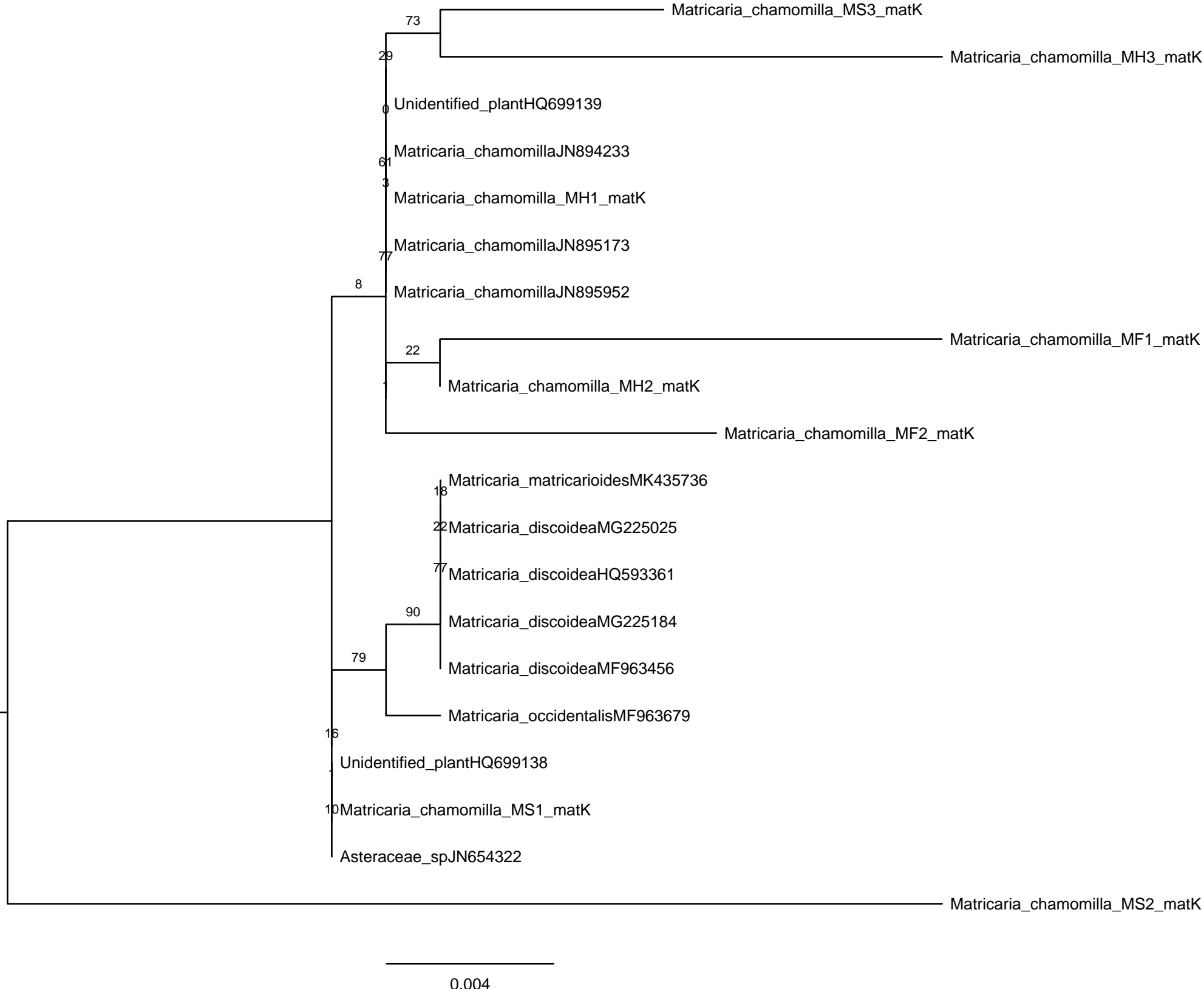

Supplement: Supplementary file 1 [file plants-09-01601-s001.zip › Figure S1.pdf]

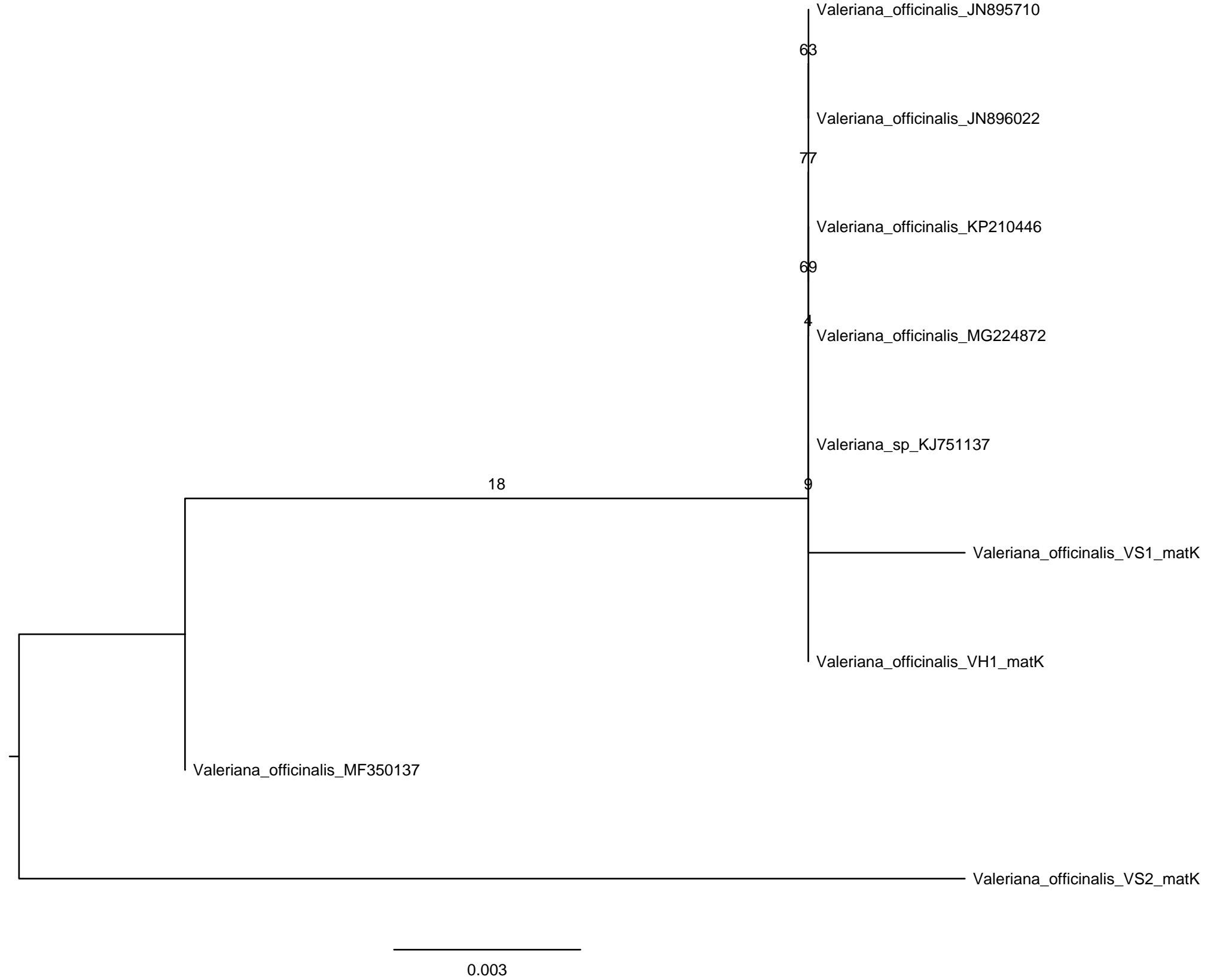

Supplement: Supplementary file 1 [file plants-09-01601-s001.zip › Figure S2.pdf]

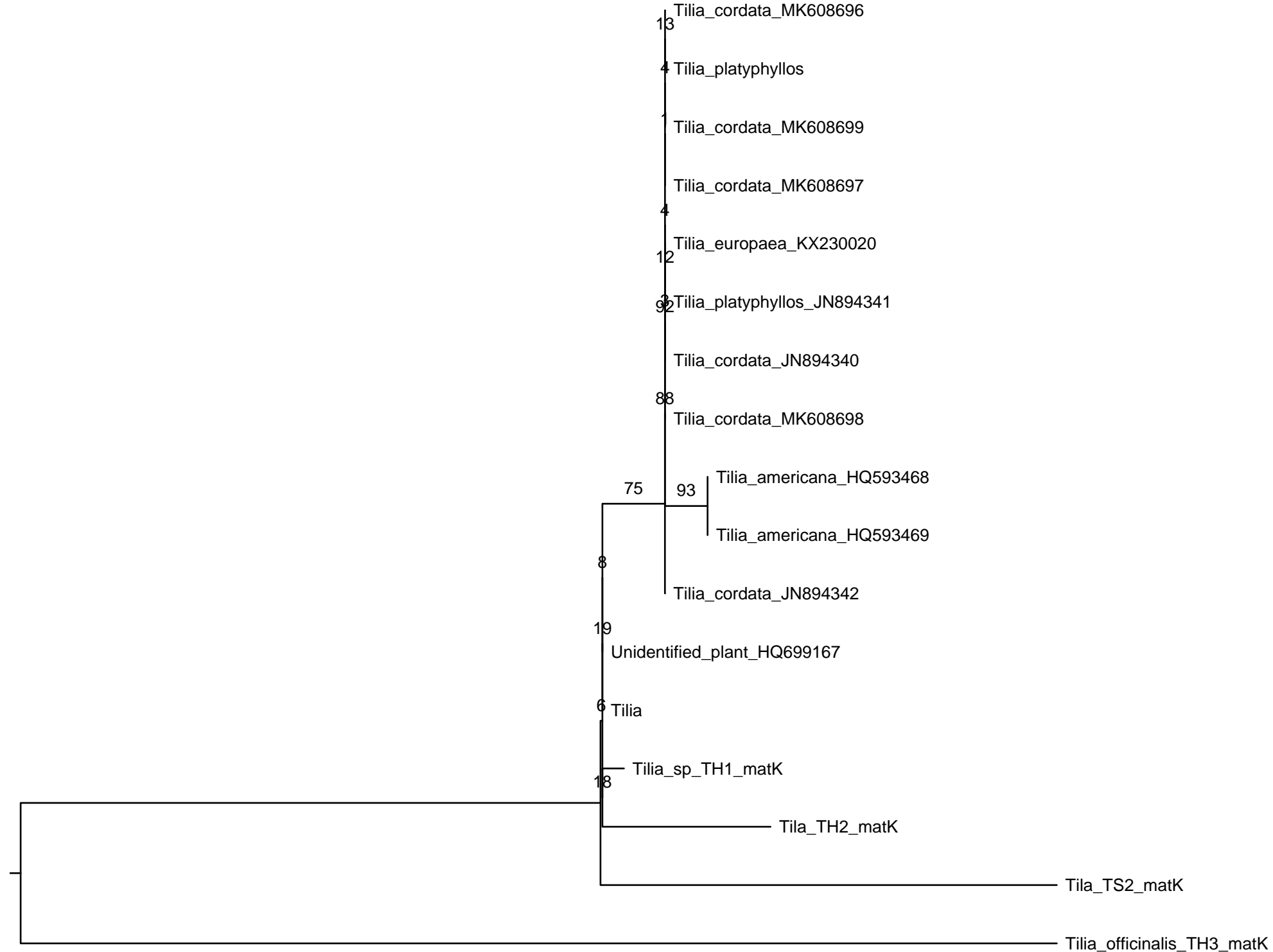

0.01

Supplement: Supplementary file 1 [file plants-09-01601-s001.zip › Figure S3.pdf]

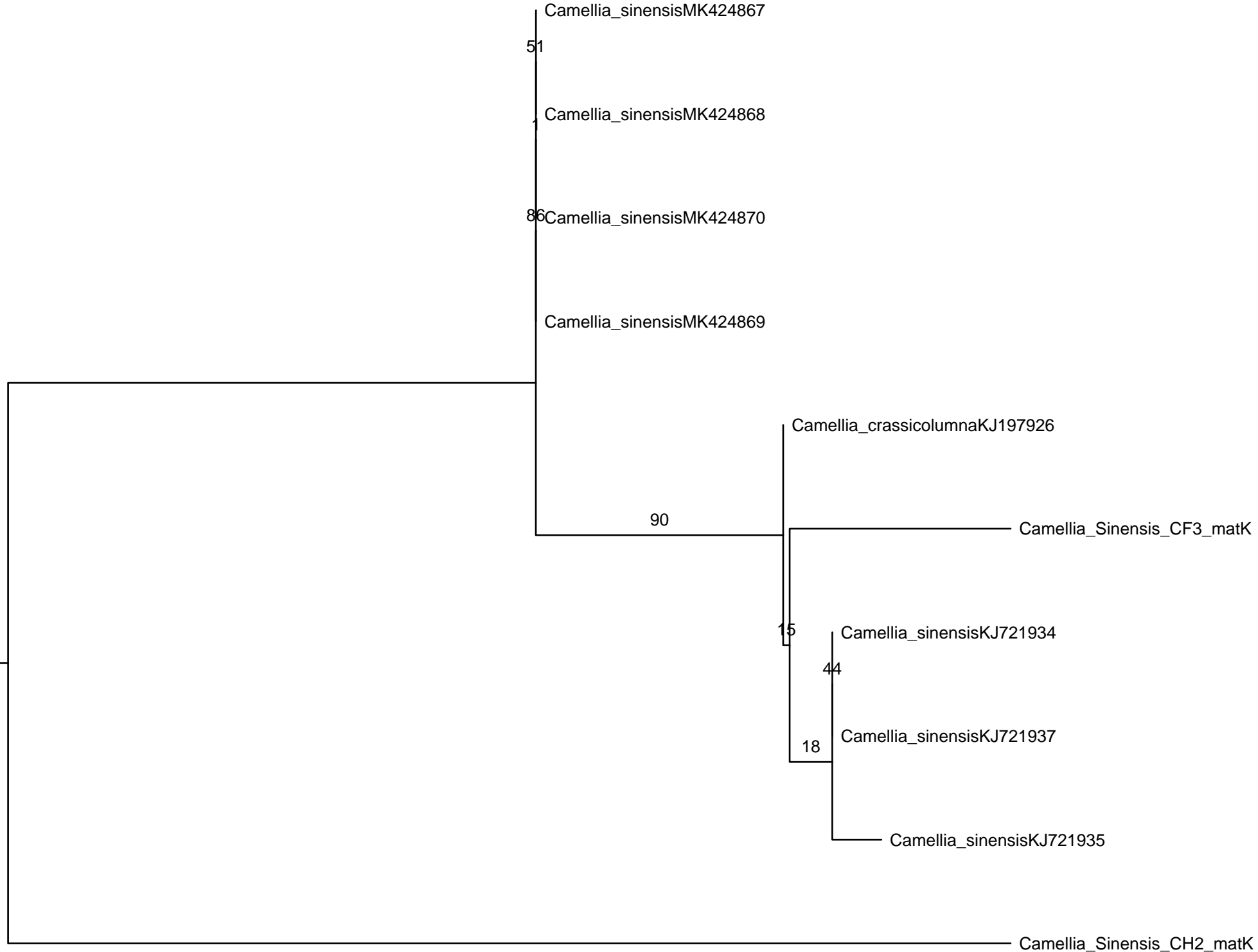

0.006

Supplement: Supplementary file 1 [file plants-09-01601-s001.zip › Figure S4.pdf]
